# The sum of absolute circadian shifts: Questioning the metric linking daylight saving time policy to stroke and obesity

**DOI:** 10.1073/pnas.2532075123

**Published:** 2026-04-17

**Authors:** José María Martín-Olalla, Jorge Mira

**Affiliations:** ^a^Facultad de Física, Departamento de Física de la Materia Condensada, Universidad de Sevilla, Sevilla ES41012, Spain; ^b^Facultade de Física, Departamento de Física Aplicada and Instituto de Materiais, Universidade de Santiago de Compostela, Santiago de Compostela ES15782, Spain

In the past years, the seasonal clock policy (seasonal Daylight Saving Time) has garnered significant attention across scientific, societal, and political spheres. A recent study sought to quantify the impact of the time policy, claiming to link US “county-level solar light patterns, time policy and health data with circadian models (1)” Authors’ key conclusion was that “permanent Standard Time (ST) would lead to a decrease in the prevalence of stroke and obesity.” With a potential reported impact on millions of persons, the study quickly found its way into social media, news outlets, blogs, and Wikipedia pages.

The study’s methodology estimates core body temperature CBT every *δ* = 5 min (1/288 of a day) across one year. Then the time of the daily minimum CBT_min_ is determined. The subsequent step was to compute the daily time difference between consecutive CBT_min_ and Earth’s rotation period (*T* = 24 h) to obtain the circadian shift $\Delta =\mathrm{diff}({\mathrm{CBT}}_{\min})-T$. The authors described the final metric—the “yearly circadian shifting”—as the sum of “all the small shifts across the year, including the one-hour shifts in the biannual shifting model, to determine overall yearly circadian burden.”

Only that, authors’ code shows that the small shifts were not actually added (2). Instead, it was the *absolute shifts* that were added to obtain the overall yearly circadian burden (see computeCircadianShifting.m lines 192 and 193 in ref. 2). That is, every shift added to the circadian burden irrespective of a delay or an advance.

**Fig. 1.**
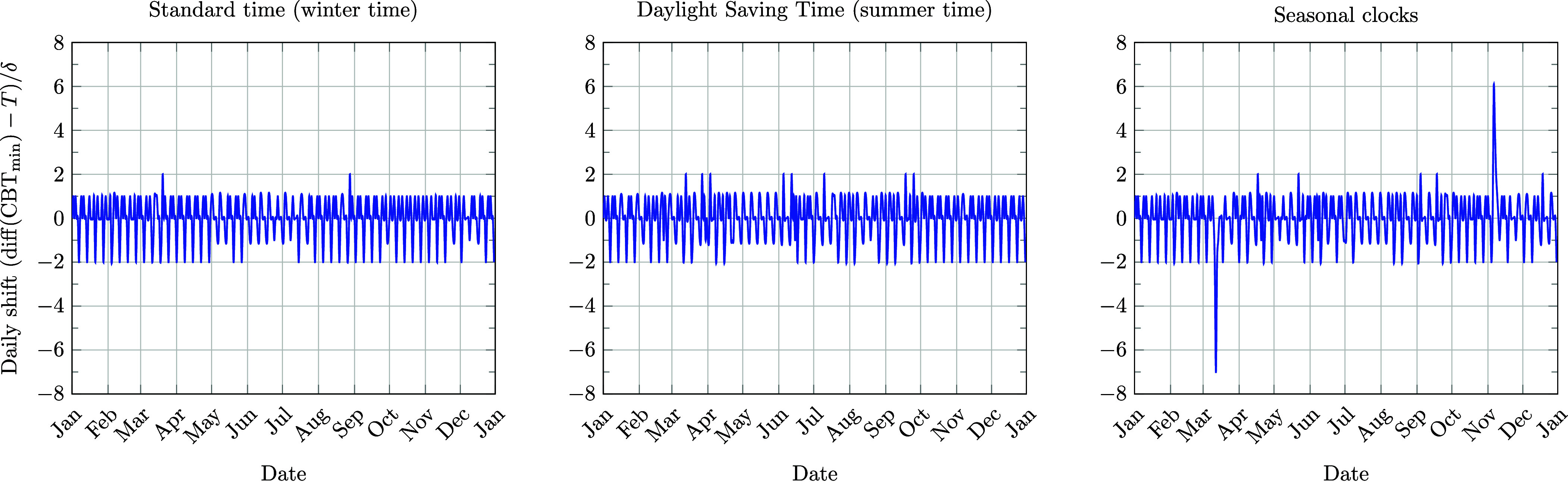
The daily circadian shift $\Delta =\mathrm{diff}({\mathrm{CBT}}_{\min})-T$ (where T=24 h) as computed using the script computeCircadianShift.m (2). Shifts are displayed on the vertical axis in units of the model’s time step, δ=5 min (1/288 of a day). The circadian period τ is set to the intermediate chronotype τ−T=2.4δ. *Left*: Permanent Standard Time (ST); *Center*: Permanent Daylight Saving Time (DST); *Right*: Current Seasonal Clocks in the United States for 2023. The location is set at San Francisco, $37.7749^{{}^{\circ}}N,122.4939^{{}^{\circ}}W$. Note the small, noisy shifts with two prominent spikes corresponding to the biannual clock transitions in the *Right* panel. In this example, for all three scenarios, the average shift is zero (∑Δ=0). SD are 0.90*δ* (*Left*), 0.95*δ* (*Center*), and 1.07*δ* (*Right*).

The core methodology might have been sound, if the authors had considered a meaningful threshold above which absolute shifts should be summed. However, authors’ code (2) shows a noisy discrete, distribution of shifts not greater than 2δ=10 min, with only significant spikes occurring at transition dates, see Fig. 1. No seasonal trends were found in the shifts, as revealed by a power spectral density analysis. In the example, the signed shifts across one year sum to zero, irrespective of the clock policy.

The seasonal clock policy is based on a natural stimulus: the varying sunrise times that seasons bring at Extratropical latitude. The magnitude of the solar swing increases with increasing absolute latitude; however, irrespective of latitude, the yearly average sunrise time is 06:00 (mean solar time). The clock policy is designed to imperfectly align working preset starting hours with these swinging sunrise times (3).

Given this context, the following critical question arises: what meaningful association with chronic health issues can be expected a priori if the sum of these absolute small shifts—a quantity that largely appears to quantify model noise—is taken as a descriptive variable?
